# Fetal ductus arteriosus constriction following maternal analgesic exposure

**DOI:** 10.1515/crpm-2026-0002

**Published:** 2026-05-13

**Authors:** Ayberk Çakır, Edis Kahraman, Deniz Gedik, Nefise Bengü Akgün, Mehmet Aytaç Yüksel

**Affiliations:** Department of Obstetrics and Gynecology, Acıbadem Mehmet Ali Aydınlar University Atakent Hospital, Istanbul, Türkiye

**Keywords:** ductus arteriosus constriction, fetal echocardiography, pregnancy, indomethacin, paracetamol

## Abstract

**Objectives:**

Premature constriction of the fetal ductus arteriosus is a clinically relevant cause of fetal cardiac compromise. Maternal exposure to prostaglandin-inhibiting analgesics is a recognized trigger. We describe two pregnancies complicated by fetal ductus arteriosus constriction temporally associated with maternal analgesic use.

**Case presentation:**

In the first case, ductal constriction occurred after a seven-day course of indomethacin initiated at 28 weeks’ gestation for polyhydramnios. Although initial improvement followed drug discontinuation, recurrent constriction with right ventricular dilation, severe tricuspid regurgitation, ductus venosus A-wave reversal, and a non-reactive fetal heart rate tracing indicated progressive hemodynamic compromise, prompting preterm delivery at 31+6 weeks. Postnatal echocardiography showed normalization of right ventricular function. In the second case, ductal constriction with right ventricular dilation and tricuspid regurgitation was detected at 38+5 weeks after frequent third-trimester use of a paracetamol-containing analgesic. Delivery at 39 weeks was performed primarily for prior uterine surgery, and postnatal evaluation confirmed normal pulmonary pressures and right ventricular function.

**Conclusions:**

Maternal exposure to prostaglandin-inhibiting analgesics should be considered in cases of fetal ductus arteriosus constriction. Careful medication history and targeted fetal echocardiography are essential for timely diagnosis and appropriate perinatal management.

## Introduction

The fetal ductus arteriosus is a critical component of normal intrauterine circulation, allowing blood to bypass the pulmonary vasculature by shunting flow from the pulmonary artery to the descending aorta. Patency during gestation is primarily maintained by prostaglandin E_2_ (PGE_2_), which induces vasodilation of ductal smooth muscle through cyclic adenosine monophosphate (cAMP)–mediated signaling and activation of EP4 receptors [1], 2]. Disruption of this prostaglandin-dependent mechanism may result in premature constriction or closure of the ductus arteriosus, leading to fetal cardiovascular compromise.

Maternal exposure to agents that inhibit cyclooxygenase (COX-1 and COX-2) is a major contributor to this disruption. Non-steroidal anti-inflammatory drugs (NSAIDs), particularly indomethacin, and less frequently paracetamol, reduce prostaglandin synthesis and have been implicated in fetal ductus arteriosus constriction [3], 4]. Resulting hemodynamic alterations may include increased right ventricular afterload, pulmonary hypertension, tricuspid regurgitation, ventricular dysfunction, and oligohydramnios due to impaired fetal renal perfusion [3]. Susceptibility of the ductus arteriosus to prostaglandin inhibition increases with advancing gestational age. After approximately 28–32 weeks, ductal patency becomes increasingly prostaglandin dependent, rendering the vessel more vulnerable to pharmacological suppression [4]. Consequently, NSAID use in late pregnancy is generally avoided or undertaken with close fetal surveillance.

Paracetamol (acetaminophen), traditionally considered a safer alternative for analgesia and antipyresis in pregnancy due to its comparatively weaker peripheral COX inhibition, has more recently been implicated in isolated reports of fetal ductal constriction [5]. Although the biological plausibility of prostaglandin suppression exists, epidemiologic evidence remains inconsistent, and a definitive causal relationship has not been established. This uncertainty is clinically relevant, as paracetamol is widely used in the third trimester and is even employed postnatally in neonates to promote ductal closure.

In this report, we present two pregnancies complicated by fetal ductus arteriosus constriction temporally associated with maternal analgesic exposure: one following documented indomethacin therapy with a dynamic course characterized by recurrence and hemodynamic compromise, and the other temporally related to paracetamol use with a more favorable outcome. By contrasting a well-established pharmacological effect with a less clearly defined association, we aim to highlight the spectrum of presentation, the importance of objective fetal echocardiographic assessment, and considerations in perinatal management.

## Case presentations

### Case 1

A 29-year-old primigravida (gravida 1, para 0) with a known diagnosis of Bartter syndrome was referred at 31+2 weeks’ gestation for evaluation of suspected fetal cardiovascular compromise. Her medical history was otherwise unremarkable, and she denied tobacco, alcohol, or illicit drug use during pregnancy. No electrolyte imbalance, renal dysfunction, or pregnancy-related complications attributable to Bartter syndrome occurred during gestation, and no intravenous electrolyte supplementation was required.

Genetic evaluation had been performed, and whole exome sequencing confirmed fetal Bartter syndrome. The pregnancy became complicated by polyhydramnios during the third trimester, and amnioreduction was performed at an external institution. The polyhydramnios was considered most likely secondary to fetal Bartter syndrome, which is known to cause fetal polyuria and increased amniotic fluid volume.

Indomethacin therapy was initiated at 28 weeks’ gestation and administered for seven consecutive days using a tapering regimen: 100 mg/day for the first three days, 75 mg on day 4, 50 mg on day 5, and 25 mg daily on days 6 and 7. The medication was subsequently discontinued. Transient echocardiographic improvement was reported between 29 and 30 weeks’ gestation.

At 31+2 weeks, evaluation at our center revealed persistent and severe polyhydramnios, with a deepest vertical pocket measuring 18 cm. Estimated fetal weight corresponded to the 14th percentile for gestational age, without evidence of fetal growth restriction. Targeted fetal echocardiography demonstrated marked constriction of the ductus arteriosus with dilation of the right atrium and right ventricle. Pulsed Doppler interrogation showed increased flow velocity across the ductus consistent with constriction; however, precise peak systolic and end-diastolic velocities and ductal diameter measurements were not retrievable from archived imaging, representing a limitation of retrospective data extraction (Supplementary Material, Video S1).

The right ventricle appeared hypertrophied and dilated with reduced systolic function. Quantitative right ventricular functional indices (ejection fraction, fractional shortening, or TAPSE) were not available; ventricular dysfunction was therefore assessed qualitatively based on chamber enlargement and visually reduced contractility. Severe tricuspid regurgitation was present, and pulmonary arterial flow could not be adequately visualized. Tricuspid regurgitation peak velocity measured 1.2 m/s. Using the simplified Bernoulli equation, the estimated tricuspid regurgitation pressure gradient was approximately 5.8 mmHg. Because fetal right atrial pressure cannot be reliably estimated, definitive right ventricular systolic pressure was not calculated.

Ductus venosus Doppler interrogation demonstrated A-wave reversal, and non-stress testing revealed a non-reactive fetal heart rate pattern. The combination of recurrent ductal constriction following drug discontinuation, right ventricular dilation with reduced systolic function, severe tricuspid regurgitation, ductus venosus A-wave reversal, and a non-reactive cardiotocographic pattern indicated progressive hemodynamic compromise.

Given the recurrence of ductal constriction after drug cessation and objective evidence of fetal circulatory decompensation, expectant management was considered unsafe. After multidisciplinary evaluation and counseling regarding the risks of prematurity vs. ongoing fetal compromise, delivery was recommended. Cesarean section was performed at 31+6 weeks’ gestation, resulting in the birth of a 1,500-g female neonate. Postnatal echocardiography demonstrated gradual normalization of right ventricular size and function, and the neonatal course was otherwise uncomplicated.

### Case 2

A 32-year-old multiparous woman (gravida 4, para 3) was referred to our center at 38+5 weeks’ gestation for targeted fetal cardiac evaluation after abnormal screening findings. The pregnancy had otherwise been uncomplicated. During the third trimester, the patient reported frequent use of an over-the-counter combination medication containing paracetamol, chlorpheniramine, and pseudoephedrine. She denied exposure to NSAIDs or other prostaglandin-inhibiting agents. Precise dosage information and serum drug levels were unavailable, limiting quantitative exposure assessment.

Routine obstetric ultrasonography demonstrated a live male fetus with appropriate growth and normal anatomical findings. Umbilical artery and middle cerebral artery Doppler indices were within normal limits. However, targeted fetal echocardiography revealed constriction of the ductus arteriosus, with a peak systolic velocity of 2.4 m/s and a ductal diameter of 2.16 mm (Supplementary Material, Video S2).

Cardiac anatomy was otherwise preserved, with a normal three-vessel view and no abnormalities of the aortic arch or left-sided cardiac structures. Nevertheless, right ventricular dilation with reduced systolic performance was observed. Moderate tricuspid regurgitation and pulmonary valve regurgitation were present. Quantitative right ventricular systolic indices (ejection fraction, fractional shortening, or TAPSE) were not available; therefore, ventricular function was assessed qualitatively based on chamber size and contractility.

Tricuspid regurgitation peak velocity measured 1.5 m/s. Using the simplified Bernoulli equation, the estimated tricuspid regurgitation pressure gradient was approximately 9 mmHg. Because fetal right atrial pressure cannot be reliably estimated, definitive right ventricular systolic pressure was not calculated and was interpreted cautiously.

Given the advanced gestational age, delivery was planned primarily due to prior uterine surgery. The presence of ductal constriction with right ventricular involvement supported timely delivery but was not the sole indication for Cesarean section. Cesarean delivery was performed at 39 weeks’ gestation, resulting in the birth of a 3,650-g male neonate. The neonatal course was uneventful, with no evidence of persistent pulmonary hypertension. Postnatal echocardiography demonstrated a patent ductus arteriosus with normalization of right ventricular size and function.

## Discussion

Premature constriction or closure of the fetal ductus arteriosus is an uncommon but clinically significant condition that may result in fetal cardiac dysfunction and neonatal morbidity. Although idiopathic cases have been described, maternal exposure to pharmacological agents that inhibit prostaglandin synthesis remains the most frequently identified and potentially preventable cause [6], 7]. The two cases presented here illustrate different clinical expressions of this condition: one associated with documented indomethacin exposure and recurrent hemodynamic compromise, and the other temporally related to paracetamol use with a milder course and favorable neonatal outcome.

Physiological patency of the fetal ductus arteriosus is maintained primarily by prostaglandin E_2_–mediated vasodilation. Inhibition of prostaglandin synthesis induces ductal smooth muscle constriction and may promote structural remodeling that can progress to irreversible narrowing or closure [8]. Hemodynamically, ductal constriction increases right ventricular afterload and pulmonary arterial pressure, which may result in right ventricular dilation or hypertrophy, tricuspid regurgitation, systolic dysfunction, and secondary pulmonary hypertension. Suppression of renal prostaglandin activity may also alter fetal renal perfusion and amniotic fluid dynamics [2].

Indomethacin is among the most extensively studied agents implicated in fetal ductal constriction. Beyond approximately 28–32 weeks of gestation, ductal patency becomes increasingly dependent on circulating prostaglandins, rendering the ductus particularly susceptible to cyclooxygenase inhibition in late pregnancy [9]. The risk of constriction appears to depend on dose, duration of exposure, and individual fetal susceptibility [10]. Although ductal narrowing may partially or fully resolve following drug discontinuation, recurrence or progression has been reported [11].

In the first case, indomethacin was administered for seven days at 28 weeks’ gestation and subsequently discontinued, with transient echocardiographic improvement observed. However, recurrent ductal constriction developed with objective evidence of hemodynamic compromise, including right ventricular dilation with reduced systolic performance, severe tricuspid regurgitation, ductus venosus A-wave reversal, and a non-reactive fetal heart rate tracing. These findings indicated circulatory decompensation despite prior withdrawal of the inciting agent. The decision for preterm delivery was therefore based on balancing the risks of prematurity against progressive fetal compromise. Postnatal echocardiography demonstrated normalization of right ventricular size and function, supporting the interpretation that the hemodynamic disturbance was reversible after delivery. Although indomethacin-associated ductal constriction is well documented, this case illustrates the potential for a dynamic course characterized by temporary reversibility followed by recurrence, emphasizing the need for continued surveillance even after medication cessation.

The presence of Bartter syndrome in this pregnancy also warrants consideration. Fetal Bartter syndrome is a recognized cause of polyhydramnios due to fetal polyuria, and in this case, the severe polyhydramnios was most plausibly attributed to this condition rather than ductal constriction itself. No maternal electrolyte or renal complications were observed during pregnancy. Thus, while Bartter syndrome likely explained the amniotic fluid abnormality, the cardiac findings were temporally associated with indomethacin exposure and recurrence following its use.

The second case highlights a less clearly established but increasingly discussed association between paracetamol exposure and fetal ductal constriction. Although paracetamol exerts weaker cyclooxygenase inhibition than NSAIDs, it may still influence prostaglandin synthesis and ductal tone, particularly with frequent or prolonged use in late gestation. Several case reports have described fetal ductal constriction temporally associated with maternal paracetamol use, with varying degrees of suspected causality [12], 13]. However, larger cohort studies have not consistently demonstrated a significant association between third-trimester paracetamol exposure and fetal cardiovascular toxicity [14]. Differences in exposure assessment, dosage, duration, and fetal susceptibility likely contribute to these discrepancies. Compared with NSAIDs, the prostaglandin-inhibiting effect of paracetamol is considerably weaker, which may explain its lower and less predictable risk profile in population-based analyses [14], 15].

In the present case, exposure assessment relied on maternal history, and precise dosage information was unavailable, limiting causal inference. Chlorpheniramine and pseudoephedrine, also contained in the reported medication, have not been associated with late-gestation ductal constriction; reported concerns primarily involve rare first-trimester malformations rather than fetal cardiovascular effects [16], [17], [18], [19], [20]. Accordingly, the observed association with paracetamol should be interpreted as temporal and biologically plausible but not definitively causal. Importantly, delivery at 39 weeks was performed primarily due to prior uterine surgery, and ductal constriction was not the sole indication for Cesarean section. Postnatal evaluation confirmed normal pulmonary pressures and normalization of right ventricular function.

Several limitations should be acknowledged. Quantitative right ventricular functional indices (ejection fraction, fractional shortening, or TAPSE) were not available in the archived fetal echocardiographic examinations, and ventricular dysfunction was assessed qualitatively. In Case 1, precise ductal peak systolic and end-diastolic velocities and ductal diameter measurements could not be retrospectively retrieved. Tricuspid regurgitation gradients were calculated using the simplified Bernoulli equation; however, because fetal right atrial pressure cannot be reliably estimated, definitive right ventricular systolic pressure values were not assigned. These limitations restrict the degree of quantitative hemodynamic analysis that can be provided.

Overall, these cases demonstrate the clinical spectrum of fetal ductus arteriosus constriction associated with prostaglandin-inhibiting analgesics, ranging from severe early-onset disease requiring preterm delivery to later-gestation presentations with favorable neonatal outcomes. While paracetamol at recommended doses is generally considered low risk, frequent or prolonged use during the third trimester warrants caution. Careful assessment of maternal medication history and consideration of targeted fetal echocardiographic evaluation may therefore be appropriate when fetal right heart abnormalities are detected.

## Conclusions

In conclusion, we described two pregnancies complicated by intrauterine ductus arteriosus constriction identified on fetal echocardiography in the setting of maternal exposure to prostaglandin-inhibiting analgesics. One fetus developed severe ductal constriction with marked right ventricular dysfunction following third-trimester indomethacin exposure, necessitating preterm delivery. The second fetus demonstrated ductal constriction with right ventricular involvement at term after frequent maternal use of a paracetamol-containing analgesic and had a favorable neonatal outcome after delivery. These observations highlight the importance of maintaining a high index of suspicion for medication-related ductal constriction when fetal right heart abnormalities are detected. Careful assessment of maternal medication history and timely targeted fetal echocardiography are essential for accurate diagnosis and appropriate perinatal management.

## Supplementary Material

Supplementary Material
