# Supplementary material for: Fetal ductus arteriosus constriction following maternal analgesic exposure
Source: Case Rep Perinat Med. 2026 May 13;15(1):20260002. doi: 10.1515/crpm-2026-0002 (PMC13168553; doi:10.1515/crpm-2026-0002)
Supplement: Supplementary file 1 — Supplementary Material [file j_crpm-2026-0002_suppl_001.docx]

**Supplementary Material**

**Supplementary Material, Video S1:** Four-chamber fetal echocardiographic view demonstrating right ventricular dilation and hypertrophy with reduced systolic contractility, consistent with right ventricular dysfunction in the setting of ductal constriction.

**Supplementary Material, Video S2:** Three-vessel trachea view demonstrating focal narrowing of the ductus arteriosus at its junction with the descending aorta. Color Doppler imaging demonstrates flow acceleration across the constricted segment, consistent with ductal constriction.
